# >The Tendency of Medico-Psychology: Freud's Theory and Its Critics

**Published:** 1914-05-16

**Authors:** 


### THE TENDENCY OF MEDICO-PSYCHOLOGY.

### Freud's Theory and its Critics.

The attention that has lately been given to the theories of Professor Freud, the well-known psychologist, by British and American alienists may be without doubt attributed to the modern tendency to study the problems of mental derangement from the psychological rather than the pathological point of view. The *post-mortem* findings in so many of the commoner forms of insanity and functional neurosis have been so meagre, that workers in this field have been glad to welcome the suggestion that possibly more rapid progress in the elucidation of the difficult problems which confront them will be made by investigating mental processes as such, without immediate reference to the organ of mind—the brain. At this juncture the painstaking researches of Freud and his co-workers offered a promising enough field, and at no previous time have the conclusions of this school been submitted to such searching tests. It is vastly important that the position to be assigned to Freudian methods in treatment, either within or without the mental hospitals, be judiciously decided.

Briefly, the basis of the new psychology is a theory that the emotions dominate human thought and conduct far more than is generally appreciated; moreover, that all the common mental disorders, including hysteria, neurasthenia, and all neuroses that do not directly depend on demonstrable organic disease, are due to emotional disturbances that have not taken a normal course. The chief abnormality that has to be considered in this respect is suppression, it being accepted that every reasonable emotion under normal circumstances must find an outlet both mental and physical. According to Freud, the suppression of an emotion results in the setting up of a focus of irritation in the mind, whence starts a long train of mental processes—which, be it noted, may be entirely outside the individual's field of thought—leading further to the development of some morbid idea, or even to physical symptoms. On this hypothesis a man who becomes very angry without being able to vent his temper by word or deed, may subsequently find himself the victim of some morbid idea or neuralgia that defies all ordinary methods of treatment. It is held that the suppression of the original anger has resulted in a storm-centre in the patient's brain, in which the emotional outburst, simmering under pressure, expresses itself in symptoms that have no apparent reference to previous events. The adherents of Freud, who appear to be steadily increasing in this country, find some such explanation sufficient to account for a host of morbid fears, unreasonable dislikes, obscure neuralgias, and other symptoms that the medico-psychologist is very familiar with.

Particularly where the trouble is periodic or paroxysmal is it believed that the theories now under consideration elucidate the problem of causation, for it is believed that the centre of irritation—the psychic trauma, as it is termed—may only be roused into activity by a recurrence of circumstances resembling those under which the original suppression of emotion occurred. Thus in a simple example cited by Freud and referred to by Dr. A. A. Brill, of New York, in his illuminating work on "Psychanalysis," a woman nursing her sick child was particularly anxious to remain silent, but, as so often happens under such circumstances, she inadvertently made a clicking noise with her tongue. Here the emotional result was to concentrate her attention on the tongue movement to a morbid degree, so that ultimately it became a habit.

Much of this work was accomplished as far back as 1895, when Freud and Breuer published a work on hysteria in which the new theories were explained; but at that time it did not appear that these investigations were likely to help the practitioner in the treatment of mental and nervous troubles, however much light they might throw on etiology. Subsequently, however, workers in this modern field of psychology found evidence suggesting that if by any means the original emotion, the suppression of which had given rise to morbid results, could be brought to the surface, as it were, and discharged, then the strain was relieved, and as a consequence the patient might be restored to the normal. The difficulty was that in many instances the original brain-storm had occurred many years previously and had completely passed out of the sufferer's memory. However, where by one means or another it was found possible to recall to mind the painful incident or impression responsible for the primary mental irritation, and the patient permitted even at that late time to give vent to some emotion, the symptoms ceased. This is the treatment called in Freudian nomenclature "catharsis," which, as defined by Brill, consists "in re-conducting the sum of excitement from its false paths to the original conscious idea, and then working it off by means of intellectual labour and speech." The mental process by which the hemmed-in emotions are discharged and the internal pressure so to say relieved is known as abreaction (*Abreagirung*), a term which signifies the mental accomplishment of something by going through the original scene in detail once more in memory. To quote Brill again, "it was noticed that the effect appeared with special intensity during the reproduction of the scenes which gave origin to the symptom and completely disappeared with their termination. On the other hand, no result was noticed when the scenes evoked were not accompanied by any emotional feeling." Another important point is that Freud and his disciples attach an overwhelming importance to one set of emotions—namely, those associated with sex; and thus it is obvious that his system of dealing with the psycho-neuroses necessitates the calling to mind of various unpleasant and not infrequently unsavoury incidents in the patient's past life which according to English notions had much better be left buried. Reading the literature of the subject, the chief features of which are readily accessible to English readers through the excellent translations and work of Brill and of Ernest Jones, it is difficult to find illustrations of the Freudian theory and method of practice which are without reference to sexual problems. It is owing to this that several of our leading psychologists have condemned the whole process, and there seems little doubt that in their original form Freud's principles will not come into general use in routine practice amongst neurologists and medico-psychological experts in this country. Originally, the exponents of the new psychology resorted to hypnotic procedures to recall the buried memories and emotions with which they wished to deal, but latterly Freud has devised a method known as psycho-analysis which is found of much greater use in the examination of thought processes. There are many who think that the chief result of his work has been the development of this process of psycho-analysis, which bids fair to become very useful in medico-psychological work apart from its original context.

